# Early Clinical Diagnosis and Treatment of Traumatic Aortic Injury Caused by Thoracic and Abdominal Injuries: A Series of Four Cases with Literature Review

**DOI:** 10.1155/2021/9995749

**Published:** 2021-04-29

**Authors:** Qiqi Wu, Shanshan Sun, Jie Xie, Tianyu Li, Hui Li, Xiangjun Bai, Zhanfei Li, Wei Wang

**Affiliations:** ^1^Department of Traumatic Surgery, Tongji Hospital, Tongji Medical College, Huazhong University of Science and Technology, Wuhan 430030, China; ^2^Department of Ultrasound Imaging Department, Tongji Hospital, Tongji Medical College, Huazhong University of Science and Technology, Wuhan 430030, China

## Abstract

Aortic injury, particularly traumatic aortic dissection caused by thoracic and abdominal injuries, is extremely rare. The diagnosis rate of blunt aortic injury caused by chest and abdominal injuries is often low, and its clinical manifestations are atypical. Once missed or misdiagnosed, the consequences are serious. Early diagnosis of traumatic aortic injury in complex thoracic and abdominal injuries is a key factor in reducing the mortality of trauma patients. Among all trauma patients treated in our department from December 2018 to December 2020, we diagnosed four cases of aortic injury, including three cases of aortic dissection and one case of intramural hematoma. Successful surgical treatment and clinical outcome were achieved in all four patients. We found that early diagnosis and surgical treatment can help to reduce the mortality of patients with traumatic aortic injury and improve the prognosis.

## 1. Introduction

Traumatic aortic injury, especially traumatic aortic dissection, has a low incidence but high lethality [1]. It is often associated with chest and abdominal injuries. It presents with atypical clinical manifestations, so it is easy to miss or misdiagnose [2, 3]. Therefore, early detection and diagnosis of traumatic aortic injury is extremely important for reducing the mortality of trauma patients and improving the prognosis. In this article, we reported four cases of traumatic aortic injury who were successfully early treated, aiming to provide a reference for their early clinical diagnosis to reduce mortality.

## 2. Case Reports

### 2.1. Case 1

**Figure 1:**
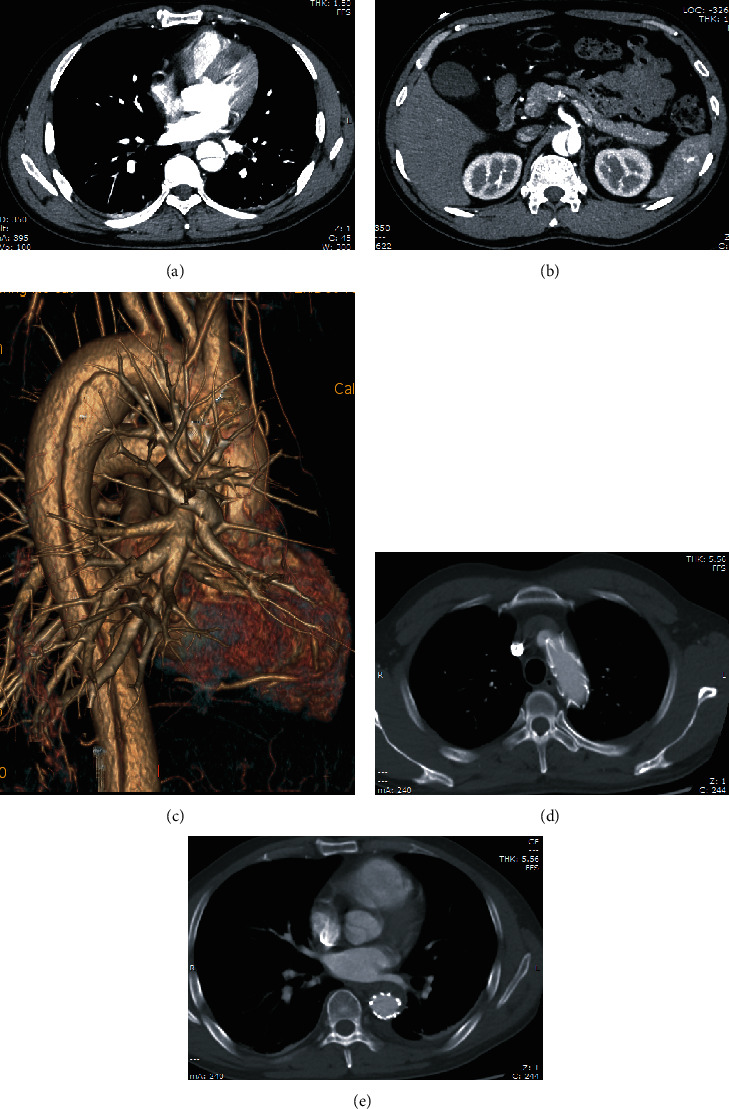
(a) CT angiography scan revealed double-lumen aortic arch structure. (b) CT angiography scan showed the double-lumen structure of the abdominal aorta. (c) CT three-dimensional imaging suggested aortic dissection. (d and e) After aortic stenting, no internal leakage was visible.

A 48-year-old man with no past significant medical illness was transferred to hospital 6 hours after the car accident. The main symptoms of the patient were chest pain, chest tightness, shortness of breath, and dyspnea. Previous chest computed tomography (CT) of the first-visit hospital indicates suspected aortic injury. Thus, CT angiography of the thoracic and abdominal aorta was performed immediately and showed type III aortic dissection. A double-lumen structure was seen in the aortic arch, descending aorta, and abdominal aorta, and the breach was located at the level of the descending aorta near the aortic arch. CT showed calcification of the aorta and coronary arteries. The diagnosis time after the injury was 8.5 hours. With the informed consent of the patient and family members, aortic stent graft placement and isolation was performed. After reexamination, there was no obvious endoleak, and the three branches of the aortic arch were normal (Figure 1).

### 2.2. Case 2

**Figure 2:**
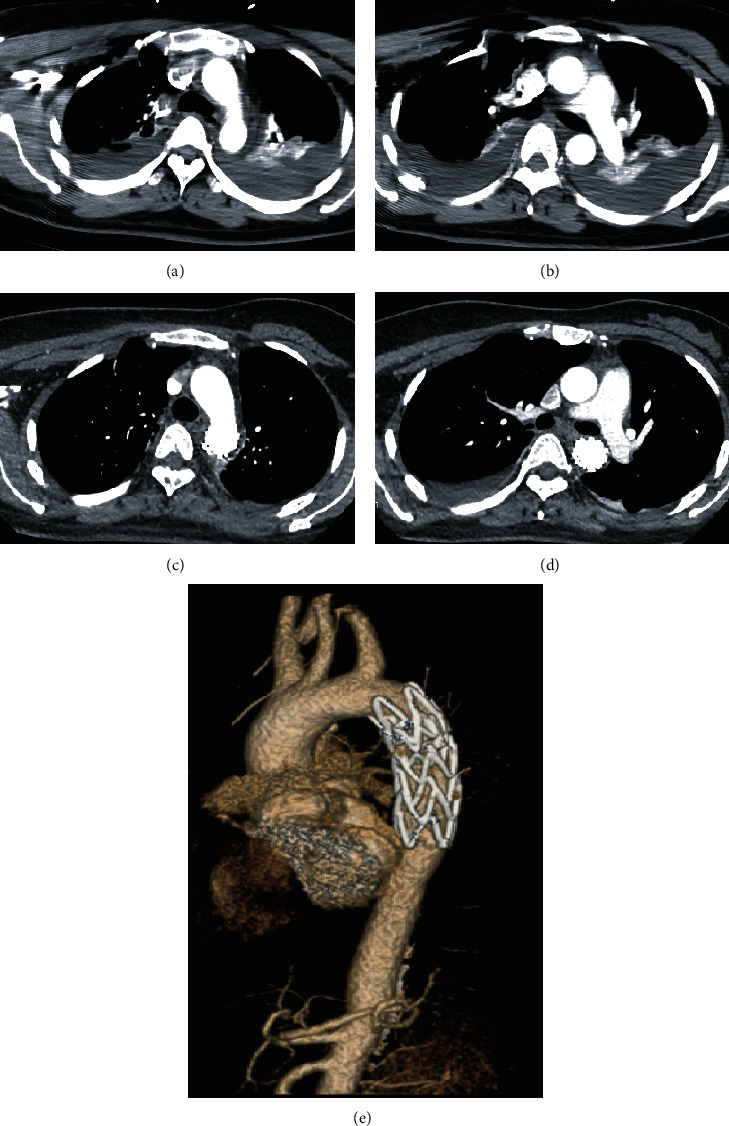
(a and b) CT angiography scan revealed double-lumen aortic arch structure. (b) Percutaneous femoral arteriography revealed a small incision with leaking of the contrast medium of the descending thoracic aorta at the level of the pulmonary artery. (d and e) After aortic stenting, no internal leakage was visible.

A 35-year-old woman was admitted to our hospital 1 hour after a traffic accident. The chief complaints were chest pain and dyspnea. The patient presented with hemorrhagic shock with a hemoglobin level of 10 g/dL and had no previous underlying disease. Pleural and pericardial effusion was found by ultrasound. With the consideration of aortic injury, CT angiography (CTA) of the thoracic and abdominal aorta was performed and revealed multiple rib fractures, bilateral pleural effusion, and atelectasis; no obvious injury was observed in the thoracic and abdominal aorta. However, the patient presented with continuous dyspnea and signs of active bleeding: (i) hypotension: systolic blood pressure fluctuated between 65 and 94 mm Hg; (ii) decrease in hemoglobin level by >2 g/dL in 2 hours. Later Doppler ultrasound revealed wider pericardial effusion and roughly the same amount of pleural effusion than the previous outcome. Then, percutaneous femoral arteriography was performed for further diagnosis and treatment. A small incision was observed that presented with leaking of the contrast medium during the examination of the descending thoracic aorta at the level of the pulmonary artery. The diagnosis time after the injury was 8 hours. Aortic stent graft placement and isolation was performed at the same time. The patient recovered very well, and no harmful events occurred during the follow-up period (Figure 2).

### 2.3. Case 3

**Figure 3:**
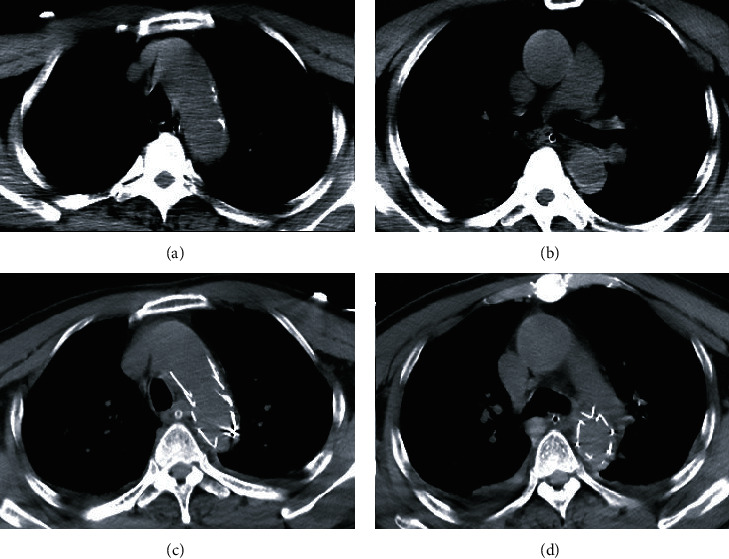
(a and b) CT angiography scan showed a suspicious crescent-shaped high-density shadow of the descending aorta. (c and d) After aortic stenting, no internal leakage was visible.

A 63-year-old man was driving alone and was involved in a traffic accident. He was transferred to our hospital 8 hours after the accident because of chest and abdominal pain. Except a history of hypertension for 10 years, he had no other specific diseases or familial medical history. Chest CT showed suspicious crescent-shaped high-density shadow of descending aorta (aortic dissection or intramural hematoma). Focused assessment with sonography for trauma (FAST) revealed no pericardial effusion. CTA examination could not be performed because the results of blood test indicated acute renal failure. After arrival, the patient quickly turned to drowsiness and unstable hemodynamic status with a progressive drop in blood pressure. Percutaneous femoral arteriography was immediately performed for hemostasis. Double-lumen structures were seen in the aortic arch, descending aorta, and abdominal aorta; three breaches were found during the examination, two of them located at the level of the descending aorta near the aortic arch and one located at the abdominal descending aorta. The diagnosis time after injury was 11 hours. With the informed consent of the patient and family members, aortic stent-graft placement and isolation was performed. Some complications such as postoperative pulmonary infection, acute liver failure, acute renal failure, and septic shock occurred in the patient. After providing respiratory support, fluid replenishment, anti-infection, and other necessary treatments, the patient died due to multiple organ failure on the 30th postoperative day. No obvious endoleak was observed during the hospital period (Figure 3).

### 2.4. Case 4

**Figure 4:**
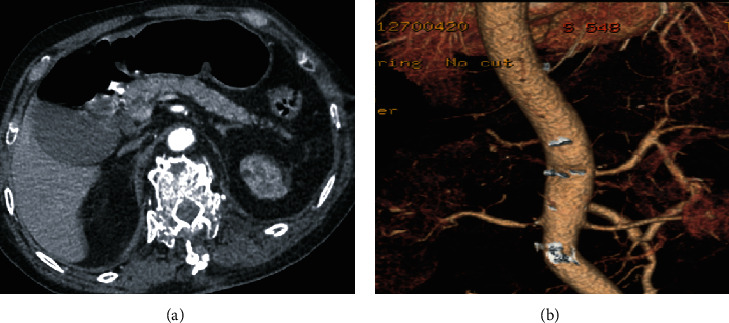
(a and b) CT angiography and three-dimensional imaging of the thoracic and abdominal aorta revealed an intramural hematoma of the abdominal aorta at the celiac trunk level.

An adult man was transferred to our emergency room 11 hours after being injured by a heavy object at the waist and back. The patient was in coma with a Glasgow Coma Scale score of 8 due to severe craniocerebral trauma. No significant past medical history was recognized. Previous chest computed tomography (CT) of the local hospital indicates suspected aortic injury. CTA of the thoracic and abdominal aorta and focused assessment with sonography for trauma (FAST) examinations revealed a 1.3 cm long intramural hematoma of the abdominal aorta at the celiac trunk level, contusion of the left kidney, and retroperitoneal hematoma. The diagnosis time after injury was 13 hours. Blood analysis showed acute liver dysfunction, acute renal failure, and myocardial injury. After comprehensive consideration of the patient's condition, medical management was performed for the treatment of the blunt aortic injury. After providing respiratory support, fluid replenishment, anti-infection, and other necessary treatments, the patient recovered very well and was discharged home on the 21st postoperative day. No obvious endoleak or other events happened until the last follow-up (Figure 4).

## 3. Discussion

Traumatic aortic injury (TAI) is a very rare disease, with an incidence of less than 1% in all trauma patients [4]. For patients with chest trauma, the incidence rate of blunt aortic injury is also extremely low. Sheehan et al. [5] have reported approximately 0.25% (1012/446950) of patients with chest trauma presented with TAI. In our trauma center, only 0.32% (4/1250) of patients associated with chest injury finally presented with TAI. However, the prognosis of TAI may be catastrophic. It is reported that more than 75% of TAI patients die before they are transferred to a medical center, and more than half of the remaining patients die within 24 hours [6]. The main causes of early death in patients with TAI include insufficient tissue perfusion and hemodynamic instability [7, 8]. With the development of imaging technology and equipment, blunt aortic injury has been detected in an increasing number of patients with abdominal or chest injury. Therefore, promoting early diagnosis and treatment plays an important role in improving the overall prognosis for TAI patients.

Unfortunately, due to the extremely low incidence, early diagnosis of TAI may be very difficult. Furthermore, TAI is often caused by high-energy injury, which usually leads to injuries in different parts of the body [9, 10]. Moar has reported that more than 80% of patients with TAI manifested as multiple injuries [11]. Williams et al. also indicated a high incidence of multiple injuries in TAI patients [12]. In this article, all patients suffered multiple injuries, which greatly increased the difficulty of early diagnosis (Table 1). One of the patients (case 4) was in coma already on admission due to severe craniocerebral injury, so we were only able to judge the existence of TAI by physical and limited imaging examinations.

Although the diagnosis of TAI is difficult, it can be assisted with diverse imaging tools, such as chest X-ray, FAST, CT, and digital subtraction angiography (DSA) [13–15]. Chest X-ray is a rapid and convenient approach for early diagnosis of aortic injury. The most significant manifestation is mediastinal widening [16]. However, chest X-ray cannot provide satisfactory sensitivity and specificity. It has been reported that approximately 7.3%–44% of patients with aortic injury present with normal mediastinum [17]. Therefore, a normal chest X-ray imaging cannot completely exclude TAI. FAST can also detect mediastinal widening rapidly and accurately with a convenient instrument. However, the sensitivity and specificity of FAST for diagnosis of patients with TAI are also very low. The gold standard for diagnosis of TAI is CT of the chest, especially CTA of the thoracic and abdominal aorta, which achieves a sensitivity of nearly 100%. Once the risk of TAI is suspected, chest CT needs to be implemented, especially for patients with chest or abdominal injuries obtained in a motor vehicle accident, even if the chest X-ray outcome is normal [18]. Although DSA can provide equal testing effectiveness as CTA of the thoracic and abdominal aorta, it is not the first choice due to its invasive nature. However, in some circumstances when CTA cannot be performed, as in the patient reported in case 3, DSA should be carried out in time to promote early diagnosis and treatment.

Apart from further imaging examinations, some other factors can assist in the early diagnosis of TAI. Lock et al. reported associated small-intestine injuries (36%), spine fractures (13%), and abdominal wall defects (10%) in patients with TAI [19]. Sheehan et al. reported that rib fractures, spine fractures, hemopneumothorax, trunk abrasion, and hypotension on admission were the risk factors of TAI. Motor vehicle accident (MVA) is the most common cause of TAI [5]. Therefore, for patients with chest or abdominal injuries, such as rib fractures, spine fractures, and small-intestine injuries, especially when they were injured in an MVA and presented with hypotension or dyspnea for hemopneumothorax on admission, enough attention should be paid to the fact that the patients may have TAI. Three of the four cases reported here were injured in MVA, and all four patients were associated with several risk factors listed above. However, due to the lack of prospective randomized controlled studies, additional well-designed high-quantity clinical trials are needed for verification.

Several grading systems of traumatic aortic injury have been proposed for clinical use, including the classic grading system of the Society for Vascular Surgery (SVS) [20], the grading system proposed by Gavant [21], and the Vancouver simplified grading system [20, 21]. The SVS classification describes four grades of TAI, ranging from intimal tear (grade I) to rupture (grade IV). Gavant described four grades, which include 7 subcategories of TAI. Similar to the SVS grading system, the Vancouver simplified grading system divides TAI into four categories, but the description is more detailed. In the cases we reported, only case 4 conformed to grade II lesion, while the remaining cases belonged to grade IV TAI (Table 2).

As for the therapeutic method for TAI, the main treatments include medical management, endovascular aortic repair (EVAR), and surgical approach [23, 24]. Open repair is a traditional treatment for severe TAI, but it has a high incidence of morbidity and mortality, especially in patients with multiple injuries [25]. Since the first report of EVAR in 1997, endoscopic repair technology has developed rapidly. EVAR has become the most popular treatment for TAI due to its high success rate, good tolerance, low morbidity, and mortality. However, the long-term prognosis of EVAR is not clear, and it is still subject to debate [26]. Some scholars believe that EVAR could be used in the vast majority of TAI patients, while others believe that EVAR should only be applicable to a specific group of patients. Finally, for grade I or grade II blunt aortic injuries, some scholars suggest that medical treatment associated with close monitoring of vital signs may be an alternative therapy to invasive repair [27]. In the cases we reported, the patient with grade II TAI in case 4 was only treated with medical management and recovered very well with an uneventful prognosis. The remaining patients, who suffered grade IV TAI, were all treated with EVAR. Except for the patient in case 3, who died of multiple organ failure secondary to pulmonary infection, all the other patients recovered well.

## 4. Conclusions

In conclusion, blunt aortic injury caused by thoracic and abdominal injuries is very rare but fatal. Therefore, promoting early diagnosis and treatment plays an important role in improving the prognosis of TAI patients. For patients who suffered chest or abdominal injuries, especially caused by high energy accidents and presented with hypotension or dyspnea on admission, we should pay enough consideration on blunt aortic injuries. Diverse imaging tools can be used for early diagnosis and treatment. Chest CT, especially CTA of the thoracic and abdominal aorta, is the gold standard for the diagnosis of TAI. DSA shows comparable sensitivity and specificity than CTA examination. Chest X-ray and FAST can also assist in the process of diagnosis with the presence of mediastinal widening. This report is helpful in deepening the understanding of blunt aortic injury, and it has important clinical significance for guiding the diagnosis and treatment of related diseases.

## Figures and Tables

**Table 1:** Characteristics of the included cases.

Cases	Age	Gender	Injury method	Grade	Time from injury to diagnosis (h)	Diagnostic tool	Injury sites	Treatment	Outcome
Case 1	48	Male	MVA	IV	8.5	CTA	Thorax	Endovascular repair	Excellent
Case 2	35	Female	MVA	IV	8	DSA	Thorax	Endovascular repair	Excellent
Case 3	63	Male	MVA	IV	11	CT	Brain, thorax	Endovascular repair	Died
Case 4	56	Male	Heavy objects injury	II	13	CTA	Spine fracture, abdomen	Medical management	Excellent

MVA: motor vehicle accident; CT: computed tomography; CTA: computed tomography angiography; DSA: digital subtraction angiography.

**Table 2:** The Vancouver simplified, Gavant, and SVS classification systems.

Grade	Vancouver simplified [22]	Gavant [21]	SVS [20]
I	Intimal flap, thrombus, or intramural hematoma < 1 cm	(a) Normal aorta, no mediastinal hematoma (b) Normal aorta, mediastinal hematoma (para-aortic)	Intimal tear
II	Intimal flap, thrombus, or intramural hematoma > 1 cm	(a) Minimal aortic injury, small (<1 cm) pseudoaneurysm, flap, or thrombus, no mediastinal hematoma (b) Minimal aortic injury, small (<1 cm) pseudoaneurysm, flap, or thrombus, mediastinal hematoma (Para-aortic)	Intramural hematoma
III	Pseudoaneurysm (simple or complex, no extravasation)	(a) >1 cm easily identified, regular, well-defined pseudoaneurysm with intimal flap or thrombus; no ascending aorta, arch, or great vessel involvement; mediastinal hematoma present (b) >1 cm easily identified, regular, well-defined pseudoaneurysm with intimal flap or thrombus; ascending aorta, arch, or great vessel involvement present; mediastinal hematoma present	Pseudoaneurysm
IV	Contrast extravasation (with or without pseudoaneurysm)	Total aortic disruption; easily identified, irregular, poorly defined pseudoaneurysm with intimal flap or thrombus; mediastinal hematoma present	Rupture

SVS: The Society for Vascular Surgery.

## Data Availability

The data for this study are available upon reasonable request to the corresponding authors.
